# The Senior Editor on His Rambles

**Published:** 1882-03

**Authors:** 


THE SENIOR EDITOR ON HIS RAMBLES.—During the past winter the senior editor of the INDEPENDENT PRACTITIONER was compelled by overwork to cease his editorial labors, and prevailed upon by his professional friends to take a trip to the sunny South. By the following extracts from a letter recently received from him, our readers will see that not only does he appreciate the beautiful and varied scenery of the flowery State, but that he also found what in large part he went in search of—an appetite. His letter is as follows:—

THE EVERETT,

JACKSONVILLE, FLORIDA, *Feb. 5*, 1881.

*Dear Dr. Rohé,*

While on my rambles I sometimes think of the cares and responsibilities of my "associates" and of my nephews in New York and shall ever be appreciative of the excellent manner in which the INDEPENDENT PRACTITIONER has been conducted in my absence. My indebtedness could be partly repaid if all of you were with me this beautiful balmy morning. From my windows, which front on Bay street, I see a florist's garden, the wharf and railroads, the schooners with their negligently furled sails, beyond which the grand old St. Johns stretches out like a gigantic mirror, with an irregular green-fringed frame arched by a cloudless sky.

While here enjoying such lovely spring-like weather, Floridian scenery, fruits, fish and game, away from the whirl of business, with all the comforts of "The Everett," a first-class hotel, one is only reminded now and then by the gentle lighting of a fly upon a bald head that this is not Paradise.

I propose to go up the St. Johns river to-morrow to enjoy fishing, hunting, scenery, oranges, etc., which the State of Flowers abundantly supplies, and report for duty by March the first.

Hoping to find a large list of subscribers since my long absence—Christmas day—and that my "associates" will indulge me in my winter's retreat a while longer, I am

Sincerely yours,

B. M. WILKERSON.

We hope our readers will bear with our Senior a little while longer. His "associates," who know how much he needed the holiday, certainly wish him all manner of enjoyment that the summer-land affords, and sincerely trust that he may return entirely restored to his pristine vigor, even to that bald head, upon which the blithesome fly produces a titillating reminder of the transience of all earthly pleasures.

WE ARE GLAD to announce that the *Scientific American* came out of the fire in New York, like the fabled Phœnix, with renewed life. The subscription lists, account books, patent records, patent drawings and correspondence were preserved in massive fire-proof safes. The printing of the *Scientific American* and *Supplement* was done in another building; consequently the types, plates, presses, paper, etc, were unharmed, and no interruption of business was occasioned.

